# Transcriptome analysis of food habit transition from carnivory to herbivory in a typical vertebrate herbivore, grass carp *Ctenopharyngodon idella*

**DOI:** 10.1186/s12864-015-1217-x

**Published:** 2015-01-22

**Authors:** Shan He, Xu-Fang Liang, Ling Li, Jian Sun, Zheng-Yong Wen, Xiao-Yan Cheng, Ai-Xuan Li, Wen-Jing Cai, Yu-Hui He, Ya-Ping Wang, Ya-Xiong Tao, Xiao-Chen Yuan

**Affiliations:** Key Laboratory of Freshwater Animal Breeding, Ministry of Agriculture, College of Fisheries, Huazhong Agricultural University, Hubei Collaborative Innovation Center for Freshwater Aquaculture, 430070 Wuhan, China; State Key Laboratory of Freshwater Ecology and Biotechnology, Institute of Hydrobiology, Chinese Academy of Sciences, 430072 Wuhan, China; Department of Anatomy, Physiology, and Pharmacology, College of Veterinary Medicine, Auburn University, Auburn, AL 36849-5519 USA

**Keywords:** Food habit transition, Carnivory, Herbivory, Grass carp, Transcriptome sequencing

## Abstract

**Background:**

Although feeding behavior and food habit are ecologically and economically important properties, little is known about formation and evolution of herbivory. Grass carp (*Ctenopharyngodon idella*) is an ecologically appealing model of vertebrate herbivore, widely cultivated in the world as edible fish or as biological control agents for aquatic weeds. Grass carp exhibits food habit transition from carnivory to herbivory during development. However, currently little is known about the genes regulating the unique food habit transition and the formation of herbivory, and how they could achieve higher growth rates on plant materials, which have a relatively poor nutritional quality.

**Results:**

We showed that grass carp fed with duckweed (modeling fish after food habit transition) had significantly higher relative length of gut than fish before food habit transition or those fed with chironomid larvae (fish without transition). Using transcriptome sequencing, we identified 10,184 differentially expressed genes between grass carp before and after transition in brain, liver and gut. By eliminating genes potentially involved in development (via comparing fish with or without food habit transition), we identified changes in expression of genes involved in cell proliferation and differentiation, appetite control, circadian rhythm, and digestion and metabolism between fish before and after food habit transition. Up-regulation of *GHRb*, *Egfr*, *Fgf*, *Fgfbp1*, *Insra*, *Irs2*, *Jak*, *STAT*, *PKC*, *PI3K* expression in fish fed with duckweed, consistent with faster gut growth, could promote the food habit transition. Grass carp after food habit transition had increased appetite signal in brain. Altered expressions of *Per*, *Cry*, *Clock*, *Bmal2*, *Pdp*, *Dec* and *Fbxl3* might reset circadian phase of fish after food habit transition. Expression of genes involved in digestion and metabolism were significantly different between fish before and after the transition.

**Conclusions:**

We suggest that the food habit transition from carnivory to herbivory in grass carp might be due to enhanced gut growth, increased appetite, resetting of circadian phase and enhanced digestion and metabolism. We also found extensive alternative splicing and novel transcript accompanying food habit transition. These differences together might account for the food habit transition and the formation of herbivory in grass carp.

**Electronic supplementary material:**

The online version of this article (doi:10.1186/s12864-015-1217-x) contains supplementary material, which is available to authorized users.

## Background

Although there are intensive research efforts on feeding behavior and food habit, little is known about the formation and evolution of herbivory. Previous studies have reported that the gut microbiota in herbivores play an important role in nutrient digestion and assimilation [1-3]. Sullam et al. suggest that herbivorous fish and mammals share the process of gut fermentation to obtain nutrients from plants [4]. However, many herbivorous fishes display low levels of gastrointestinal fermentation [5]. Several freshwater herbivorous fishes such as grass carp do not rely on microbial cellulolysis, but rather pass large quantities of plant material rapidly through the gut [6-9]. Little is known about the molecular mechanism of the formation of herbivory. Grass carp (*Ctenopharyngodon idella*) is an ecologically appealing model of vertebrate herbivore, widely cultivated in China as well as in many other countries as edible fish or as biological control agents for aquatic weeds. Grass carp goes through a transition from carnivory to herbivory during its life cycle. Grass carp smaller than 3 cm (total length) is carnivorous, fish of 3-5.5 cm (total length) is at the food transition stage from zooplankton or benthos to aquatic macrophytes, whereas fish lager than 5.5 cm (total length) is herbivorous [10]. However, little is currently known about genes determining the food habit transition, and how they could achieve higher growth rates on plant materials, which have a relatively poor nutritional quality. Therefore, grass carp is an excellent model for studying the formation mechanism of herbivory as it shows the food habit transition from carnivory to herbivory. It could facilitate the comparison analysis between carnivory and herbivory in one species, eliminating the differences result from different species.

Grass carp is not able to synthesize cellulase enzyme, and its intestinal microbiota harbors many cellulose-decomposing bacteria [11]. The cellulase enzymes produced by cellulolytic bacteria and fungi are active in a wide range of invertebrate taxa [12,13]. However, relatively few higher vertebrates are able to utilize this resource efficiently [14]. Moreover, our previous study suggests that the cellulase enzyme synthesized by certain microorganism is too limited to digest and absorb crude fiber sufficiently in grass carp, and exogenous cellulase needs to be added to the artificial diets, especially when using plant ingredients [15]. This agrees well with the results that digestion of fiber in grass carp is incomplete, with about half the food material ingested excreted as feces [16]. Therefore, the food habit transition of grass carp might be not attributed to intestinal cellulose-decomposing bacteria, but rather due to high feeding rates. It has been reported that the daily ration (the relation of the total weight of feed taken in a day to the weight of the fish) of grass carp may reach 49.9% when feeding on aquatic plants [17]. Cui et al. [18] also found that grass carp fed with plant diet spend longer time on feeding, have higher feeding intensities, and consume less dry matter per bite than those fed with animal diet. Grass carp fed with plant diet feed almost continuously for most of the diet cycle. In addition, our previous study reported that the gut length relative to body length in grass carp fed with duckweed is higher than those fed with chironomid larvae [19], suggesting that gut growth could be also involved in the food habit transition.

To elucidate the relationship between gene expression and the formation of herbivory, we performed transcriptome sequencing of grass carp before and after the food habit transition from carnivory to herbivory. We showed that expression of genes in several pathways, including cell proliferation and differentiation, appetite control, circadian rhythm, and digestion and metabolism, were significantly different in fish before and after the food transition. These potential determinants provide a glimpse of genetic architecture of the formation of herbivory. Elucidating the genes regulating the unique food habit transition from carnivory to herbivory in grass carp could lead to a better understanding of the mechanism of higher intake and utilization of plant feedstuff in grass carp or other herbivores and formation of herbivore during speciation.

## Results

### Determination of morphological characteristics

**Figure 1:**
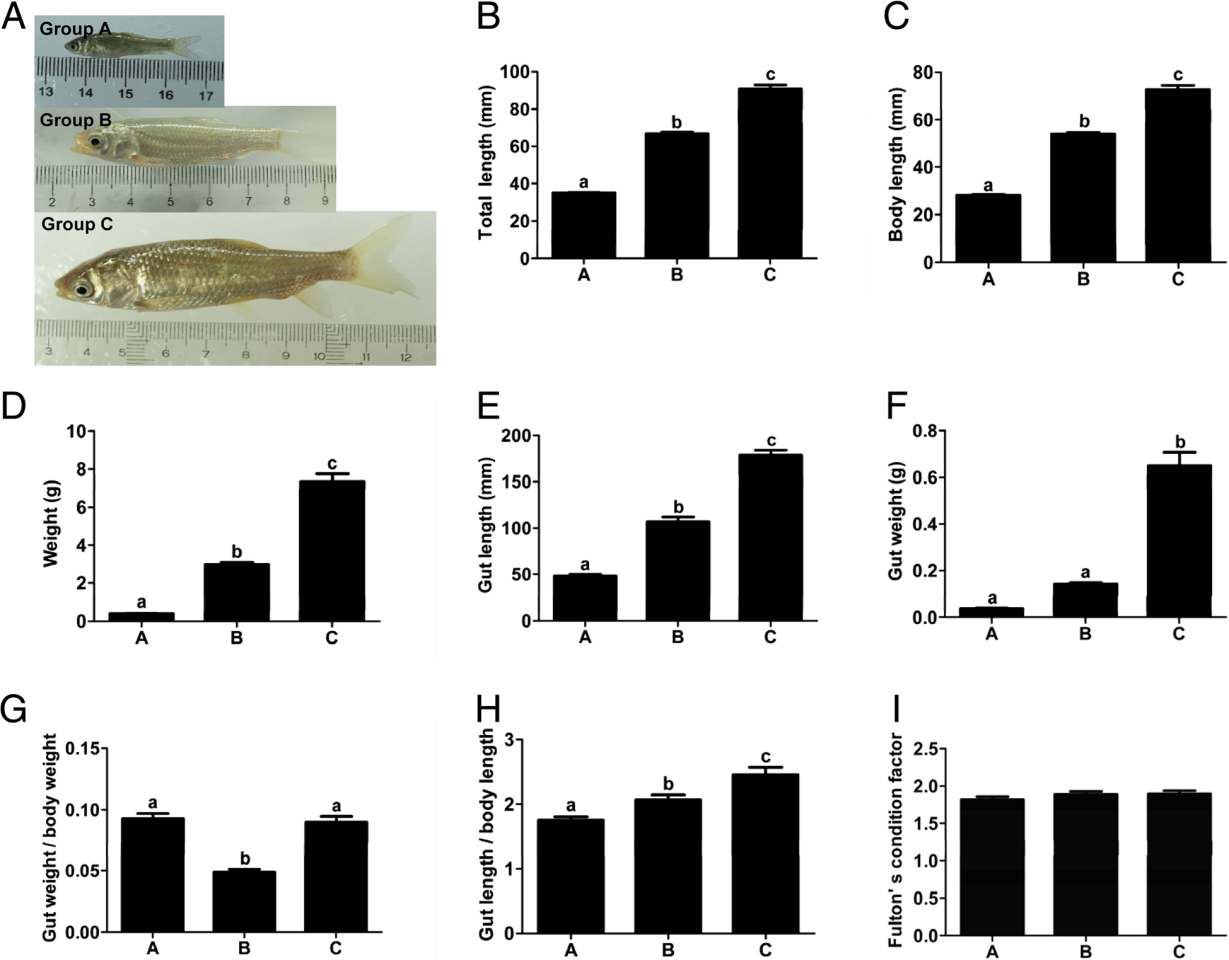
**The morphological index of grass carp with differential feeding patterns, including photos of fish (A), total length (B), body length (C), weight (D), gut length (E), gut weight (F), gut weight/body weight (G), gut length/body length (H), Fulton's condition factor (I).** Group A: fish fed with chironomid larvae before food habit transition; Group B: fish fed with chironomid larvae without transition; Group C: fish fed with duckweed after food habit transition to herbivory. Total length means the length from the rostral tip of jaw to the caudal tip of the expanded tail, and body length refers to the length from the rostral tip of jaw to the caudal end of last lateral line scale. Data are means ± S.E.M. (n = 6). A value followed by a superscript differs significantly (P < 0.05) from all other values not followed by the same superscript.

Grass carp fed with duckweed (Group C) had significantly higher relative length of gut (gut length/body length) than fish before food habit transition (Group A) or those without transition (fed with chironomid larvae) (Group B) (Figure 1). Moreover, fish fed with duckweed (Group C) had significantly higher growth than those fed with chironomid larvae (Groups B) (P < 0.05) in terms of total length, body length, gut length, body weight, gut weight (Figure 1).

### High-throughput sequencing and mapping

To obtain an overview of gene expression profile in grass carp before and after food habit transition, cDNA libraries were constructed from brain (AB), liver (AL), gut (AG) of grass carp before the food transition (before the feeding trial (Group A)); brain (BB), liver (BL), gut (BG) of fish without transition (fed with chironomid larvae (Group B)); brain (CB), liver (CL), gut (CG) of fish after food transition (fed with duckweed (Group C)), and sequenced using the Illumina HiSeq2000 system. After removing the low-quality reads, we obtained 64,914,000 (AB), 62,801,686 (AL), 62,679,236 (AG), 66,466,322 (BB), 65,442,204 (BL), 63,096,538 (BG), 62,196,672 (CB), 62,079,494 (CL), 66,338,198 (CG) clean reads, respectively (Table 1). About 66.32-76.59% of clean reads could be mapped to grass carp genome and 30.60-55.24% mapped to gene (Table 1, Additional file 1). Most of the mapped reads were perfect match (46.72-58.74% and 24.07-42.81% of clean reads to genome and genes, respectively). The vast majority of all reads were mapped to unique positions except a very small percentage (1.69-3.09%). To evaluate the quality of the transcriptomes, sequencing randomness assessment was performed to detect the random distribution of reads in reference genes. The distributions of reads from nine samples were homogeneous in grass carp genes, suggesting that the quality of our sequencing data was good. The distributions of genes’ coverage were shown by pie charts in the electronic supplementary material, Additional file 2. A large percentage of genes (26-50%) showed perfect coverage (90-100%). Most of genes’ coverage (65-83%) was higher than 50%. Overall, high quality sequencing and mapping results were obtained. The sequencing data in this study have been deposited in the Short Read Archive (SRA) at the National Center for Biotechnology Information (NCBI) (accession number: SRX532425, SRX532426, SRX532427, SRX532428, SRX532455, SRX532456, SRX532457, SRX532459 and SRX532460).

**Table 1:** **Summary of data generated from grass carp transcriptome**

**Summary**		**A**			**B**			**C**		
		**Brain**	**Gut**	**Liver**	**Brain**	**Gut**	**Liver**	**Brain**	**Gut**	**Liver**
Map to genome	Total Reads	64914000 (100.00%)	62679236 (100.00%)	62801686 (100.00%)	66466322 (100.00%)	63096538 (100.00%)	65442204 (100.00%)	62196672 (100.00%)	66338198 (100.00%)	62079494 (100.00%)
	Total BasePairs	5842260000 (100.00%)	5641131240 (100.00%)	5652151740 (100.00%)	5981968980 (100.00%)	5678688420 (100.00%)	5889798360 (100.00%)	5597700480 (100.00%)	5970437820 (100.00%)	5587154460 (100.00%)
	Total Mapped Reads	49715390 (76.59%)	42833150 (68.34%)	42985997 (68.45%)	50210581 (75.54%)	43776373 (69.38%)	43400816 (66.32%)	47148316 (75.81%)	45696711 (68.88%)	43017860 (69.29%)
	Perfect match	37846054 (58.30%)	31528381 (50.30%)	30706529 (48.89%)	37491544 (56.41%)	31800339 (50.40%)	30572108 (46.72%)	36533605 (58.74%)	33008461 (49.76%)	31336236 (50.48%)
	≤5 bp mismatch	11869336 (18.28%)	11304769 (18.04%)	12279468 (19.55%)	12719037 (19.14%)	11976034 (18.98%)	12828708 (19.60%)	10614711 (17.07%)	12688310 (19.13%)	11681624 (18.82%)
	Unique match	48342500 (74.47%)	40897483 (65.25%)	41471932 (66.04%)	48691487 (73.26%)	41959590 (66.50%)	42291590 (64.62%)	45938250 (73.86%)	43731437 (65.92%)	41795927 (67.33%)
	Multi-position match	1372890 (2.11%)	1935667 (3.09%)	1514065 (2.41%)	1519094 (2.29%)	1816783 (2.88%)	1109226 (1.69%)	1210066 (1.95%)	1965334 (2.96%)	1221933 (1.97%)
	Total Unmapped Reads	15198610 (23.41%)	19846086 (31.66%)	19815689 (31.55%)	16255741 (24.46%)	19320165 (30.62%)	22041388 (33.68%)	15048356 (24.19%)	20641427 (31.12%)	19061634 (30.71%)
Map to gene	Total Reads	64914000 (100.00%)	62679236 (100.00%)	62801686 (100.00%)	66466322 (100.00%)	63096538 (100.00%)	65442204 (100.00%)	62196672 (100.00%)	66338198 (100.00%)	62079494 (100.00%)
	Total BasePairs	5842260000 (100.00%)	5641131240 (100.00%)	5652151740 (100.00%)	5981968980 (100.00%)	5678688420 (100.00%)	5889798360 (100.00%)	5597700480 (100.00%)	5970437820 (100.00%)	5587154460 (100.00%)
	Total Mapped Reads	19866103 (30.60%)	29656785 (47.32%)	31817288 (50.66%)	27628167 (41.57%)	26090271 (41.35%)	35023773 (53.52%)	26422927 (42.48%)	30446461 (45.90%)	34294871 (55.24%)
	Perfect match	15622780 (24.07%)	22768765 (36.33%)	24089872 (38.36%)	21432825 (32.25%)	19713788 (31.24%)	26347072 (40.26%)	21345319 (34.32%)	22979918 (34.64%)	26576843 (42.81%)
	≤4 bp mismatch	4243323 (6.54%)	6888020 (10.99%)	7727416 (12.30%)	6195342 (9.32%)	6376483 (10.11%)	8676701 (13.26%)	5077608 (8.16%)	7466543 (11.26%)	7718028 (12.43%)
	Unique match	19376349 (29.85%)	28694650 (45.78%)	30598309 (48.72%)	26605613 (40.03%)	25430799 (40.30%)	34464752 (52.66%)	25908582 (41.66%)	29607406 (44.63%)	33598282 (54.12%)
	Multi-position match	489754 (0.75%)	962135 (1.54%)	1218979 (1.94%)	1022554 (1.54%)	659472 (1.05%)	559021 (0.85%)	514345 (0.83%)	839055 (1.26%)	696589 (1.12%)
	Total Unmapped Reads	45047897 (69.40%)	33022451 (52.68%)	30984398 (49.34%)	38838155 (58.43%)	37006267 (58.65%)	30418431 (46.48%)	35773745 (57.52%)	35891737 (54.10%)	27784623 (44.76%)

A: fish fed with chironomid larvae before food habit transition; B: fish fed with chironomid larvae without transition; C: fish fed with duckweed after food habit transition to herbivory.

### Alternative splicing and novel transcript predication

**Figure 2:**
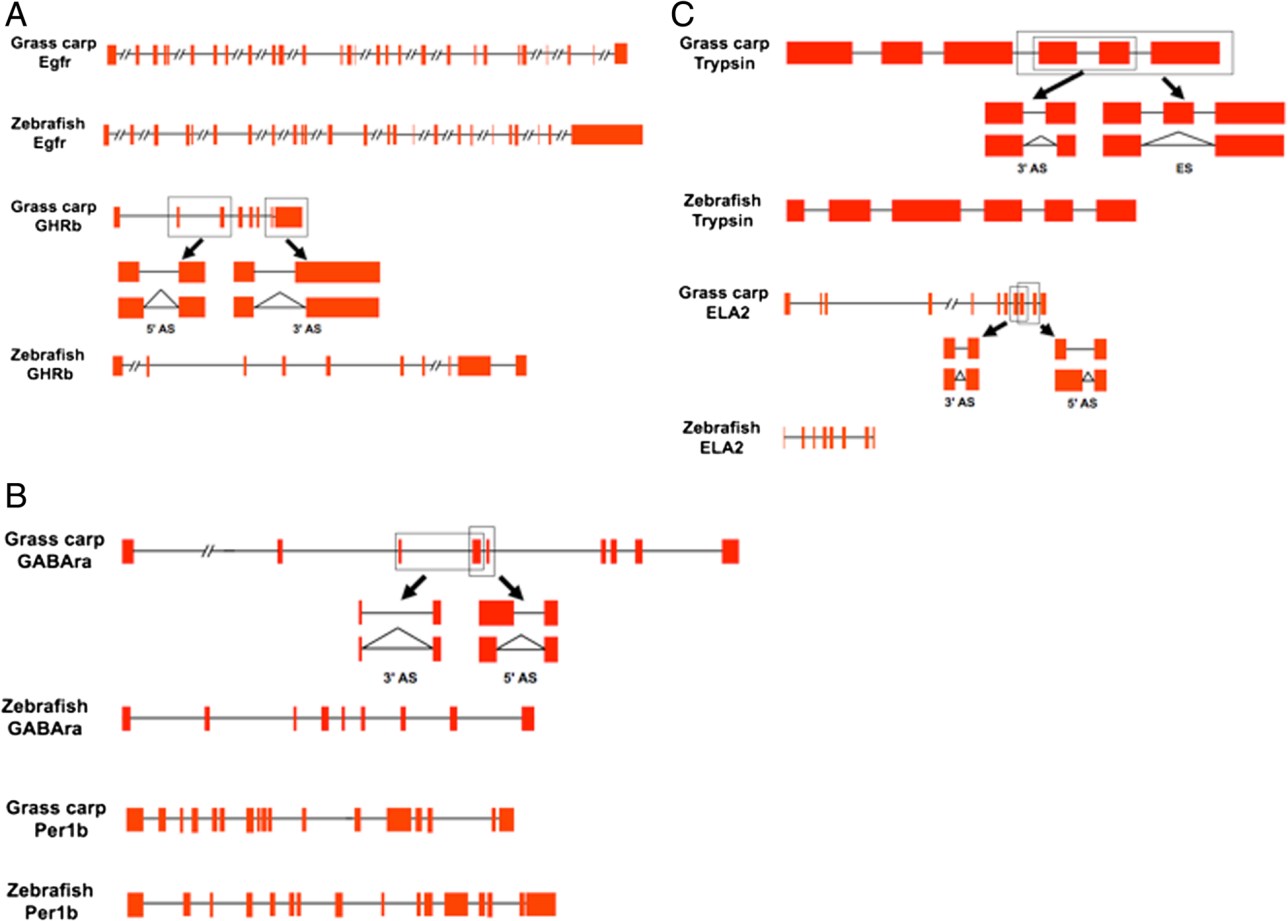
**Gene structure and alternative splicing of the most representative differentially expressed genes in cell proliferation and differentiation pathway (A), in appetite control and circadian rhythm pathway (B), in digestion and metabolism pathway (C).** The red blocks show the exons. *Egfr* means epidermal growth factor receptor, *GHRb* means growth hormone receptor b, *ELA2* means elastase 2, *GABAra* means GABA A receptor, and *Per1b* means period 1b. ES, 5’AS and 3’AS mean exon skipping, alternative 5’ splicing, and alternative 3’ splicing, respectively.

Alternative splicing is essential for protein diversity and functional complexity [20,21]. We examined four major alternative splicing events of each group, including exon skipping, intron retention, alternative 5’ splicing and alternative 3’ splicing. 15,739, 16,380 and 16,826 alternative splicing events were identified in Groups A, B and C, respectively (Additional file 3*a*). As in other vertebrates, 5’- and 3’- alternative splicing is the major class accounting for about 79.3%, 84.2% and 85.9% (12,480 in Group A, 13,790 in Group B and 14,450 in Group C) of all alternative splicing events in grass carp. Because some genes produced two or more alternative splicing events, a total of 8,859 genes (6,246 in Group A, 6,251 in Group B and 6,327 in Group C) were estimated to undergo alternative splicing (Additional file 3*a*). We found the number of exon skipping, alternative 5’ splicing and alternative 3’ splicing in Group C was higher than those in Groups A and B, whereas the number of intron retention in Group C was lower than those in Groups A and B (Additional file 3*b*). We also found 115 food habit transition-specific alternative splicing genes, involved in cell proliferation and differentiation, appetite control, circadian rhythm, mitogen-activated protein kinases (MAPK) signaling, adipocytokine, glutamatergic synapase, calcium signaling, GABAergic synapase, insulin signaling, peroxisome proliferator activated receptors (PPAR) signaling, pancreatic secretion, protein digestion and absorption, bile secretion and gastric acid secretion pathways. We suggested that these genes with alternative splicing might play important roles in the food habit transition of grass carp through regulating diverse pathways (Figure 2).

In addition, novel transcript could be determined by highthroughput sequencing to enrich the present genome database. We predicted 69,520, 43,953, 26,592, 49,109, 41,754, 16,897, 46,457, 36,675 and 18,331 novel transcripts in AB, AG, AL, BB, BG, BL, CB, CG and CL, respectively. Of these about 14.5, 15.6, 11.1, 16.6, 15.4, 13.7, 17.5, 15.1 and 14.4% (10,078 in AB, 6,870 in AG, 2,942 in AL, 8,176 in BB, 6,417 in BG, 2,313 in BL, 8,109 in CB, 5,540 in CG and 2,641 in CL) were longer than 500 bp. In all three tissues, more novel transcripts were identified in Group A than in Groups B and C, suggesting that novel transcripts were developmentally regulated.

#### Identification of differentially expressed genes

**Figure 3:**
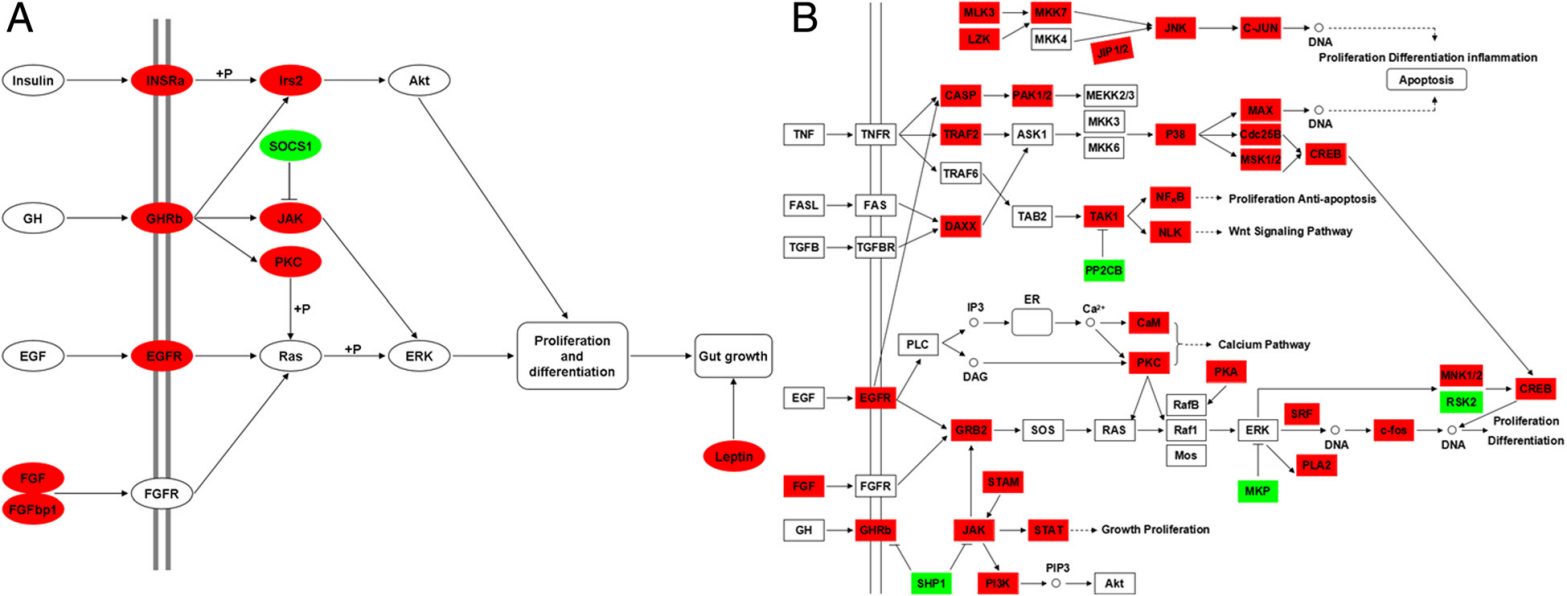
**Differentially expressed genes in cell proliferation and differentiation pathway between grass carp before and after food habit transition from transcriptome analysis.** The most important pathways are cell proliferation and differentiation **(A)**, MAPK signaling **(B)**. The colors of ellipses or rectangles were shaded according to the different expression (red: the mRNA expression levels of fish in Group C were significantly higher than those in Group A (FDR ≤ 0.001, the absolute value of log2[Ratio] ≥ 1); green: the mRNA expression levels of fish in Group C were significantly lower than those in Group A (FDR ≤ 0.001, the absolute value of log2[Ratio] ≥ 1)). All of these genes were not differentially expressed between Groups A and B.

**Figure 4:**
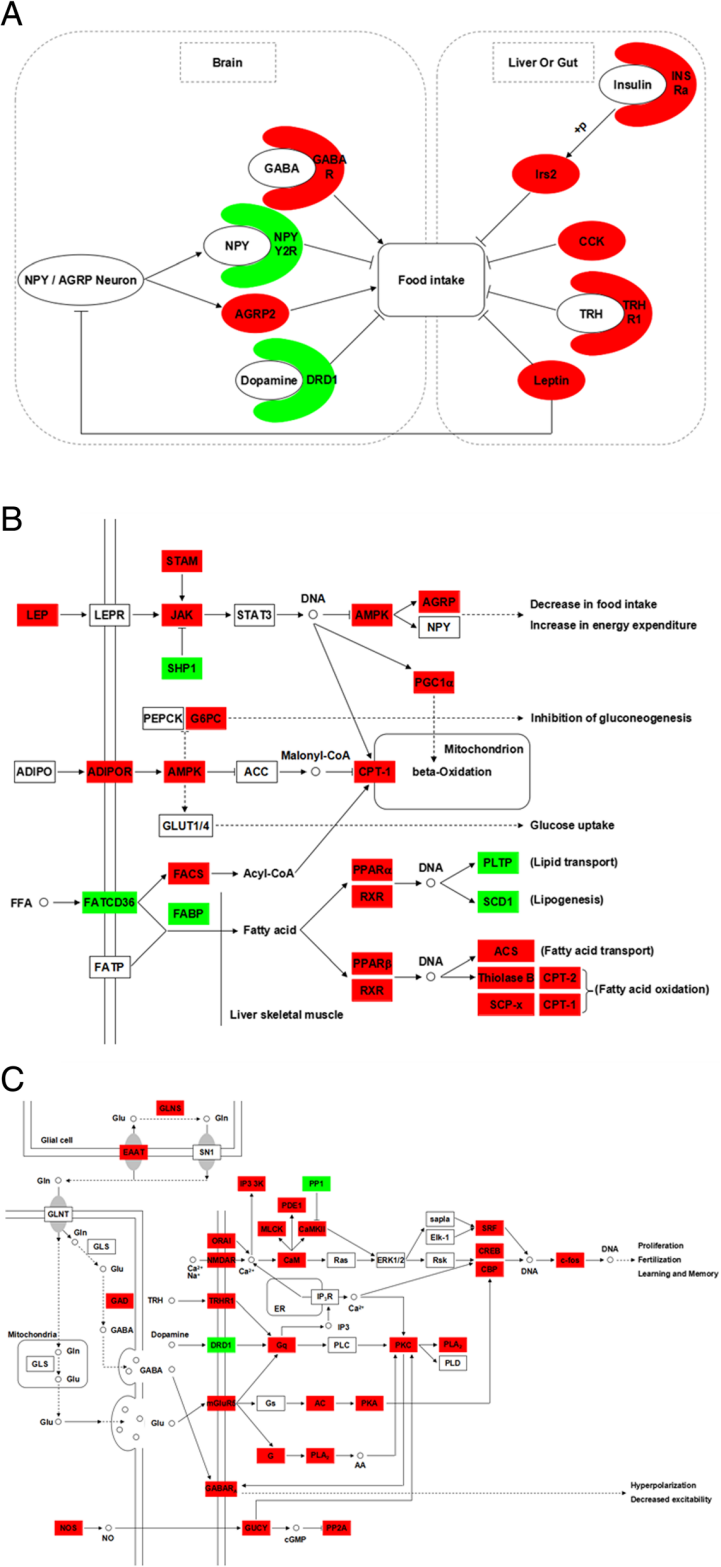
**Differentially expressed genes in appetite control pathway between grass carp before and after food habit transition from transcriptome analysis.** The most important pathways are appetite control **(A)**, adipocytokine signaling **(B)**, glutamatergic synapase, calcium signaling and GABAergic synapase **(C)**. The colors of ellipses or rectangles were shaded according to the different expression (red: the mRNA expression levels of fish in Group C were significantly higher than those in Group A (FDR ≤ 0.001, the absolute value of log2[Ratio] ≥ 1); green: the mRNA expression levels of fish in Group C were significantly lower than those in Group A (FDR ≤ 0.001, the absolute value of log2[Ratio] ≥ 1)). All of these genes were not differentially expressed between Groups A and B.

**Figure 5:**
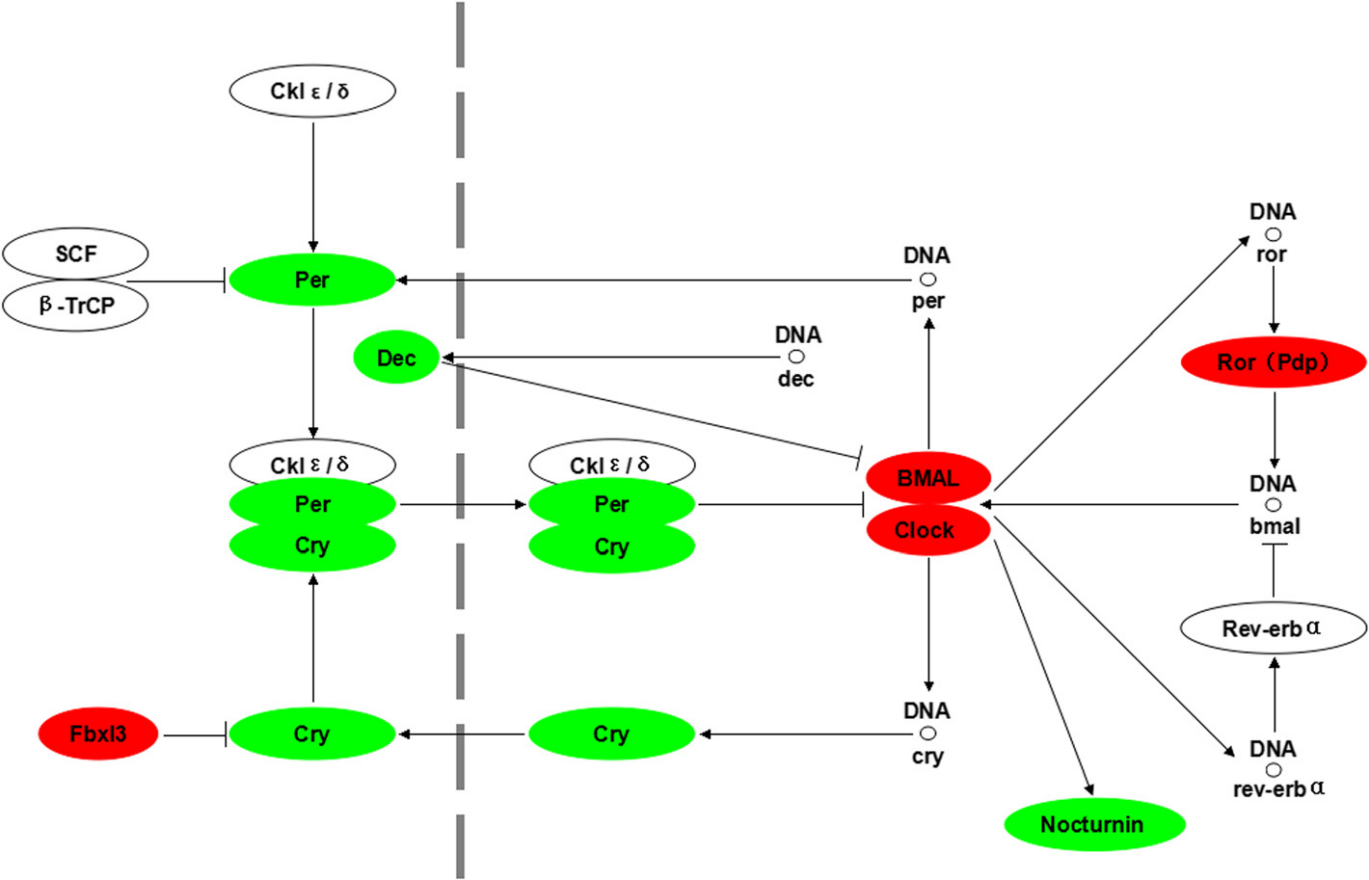
**Differentially expressed genes in circadian rhythm pathway between grass carp before and after food habit transition from transcriptome analysis.** The colors of ellipses were shaded according to the different expression (red: the mRNA expression levels of fish in Group C were significantly higher than those in Group A (FDR ≤ 0.001, the absolute value of log2[Ratio] ≥ 1); green: the mRNA expression levels of fish in Group C were significantly lower than those in Group A (FDR ≤ 0.001, the absolute value of log2[Ratio] ≥ 1)). All of these genes were not differentially expressed between Groups A and B.

We found 10,184 genes to be differentially expressed between Groups A and C, 8,711 genes between Groups A and B, 4,435 genes between Groups B and C; and 40,149 genes to be differentially expressed between brain and gut, 47,849 genes between brain and liver, 35,434 genes between liver and gut (False Discovery Rate (FDR) ≤ 0.001, fold-change ≥ 2, Additional file 4). Genes differentially expressed between Groups A and C, but not differentially expressed between Groups A and B were potentially involved in the food habit transition of grass carp. We mapped the differentially expressed genes to the reference canonical pathways in Kyoto Encyclopedia of Genes and Genomes (KEGG) to identify the biological pathways. The representative pathways with the differentially expressed genes were MAPK signaling, adipocytokine, glutamatergic synapase, calcium signaling, GABAergic synapase, insulin signaling, PPAR signaling, pancreatic secretion, protein digestion and absorption, bile secretion and gastric acid secretion and mammalian circadian rhythm pathways. Analysis of these genes, which were differentially expressed between Groups A and C, but not differentially expressed between Groups A and B, revealed the signaling pathways involved, including cell proliferation and differentiation (growth hormone receptor b (*GHRb*), epidermal growth factor receptor (*Egfr*), fibroblast growth factor (*Fgf*), FGF-binding protein 1 (*Fgfbp1*), insulin receptor a (*Insra*), insulin receptor substrate 2 (*Irs2*), Janus kinase (*Jak*), signal transducer and activator of transcription 1 (*STAT*), phosphatidylinositol-4,5-bisphosphate 3-kinase (*PI3K*), protein kinase C (*PKC*), suppressor of cytokine signaling 1 (*SOCS1*)) (Figure 3); appetite control (agouti gene-related protein 2 (*Agrp2*), neuropeptide Y receptor 2 (*Npy y2*), dopamine receptor D1 (*Drd1*), GABA A receptor (*GABAra*), leptin (*Leptin*), cholecystokinin (*Cck*), insulin receptor a (*Insra*), insulin receptor substrate 2 (*Irs2*), thyrotropin-releasing hormone receptor 1 (*Trhr1*)) (Figure 4); circadian rhythm (period 1 (*Per1*), *Per3*, cryptochrome 5 (*Cry5*), *Cry2*, clock protein (*Clock*), *Bmal2*, *hepatic leukemia factor* (*Pdp*), *class B basic helix-loop-helix protein* (*Dec*), *F-box and leucine-rich repeat protein 3* (*Fbxl3*), *nocturnin*) (Figure 5).

**Figure 6:**
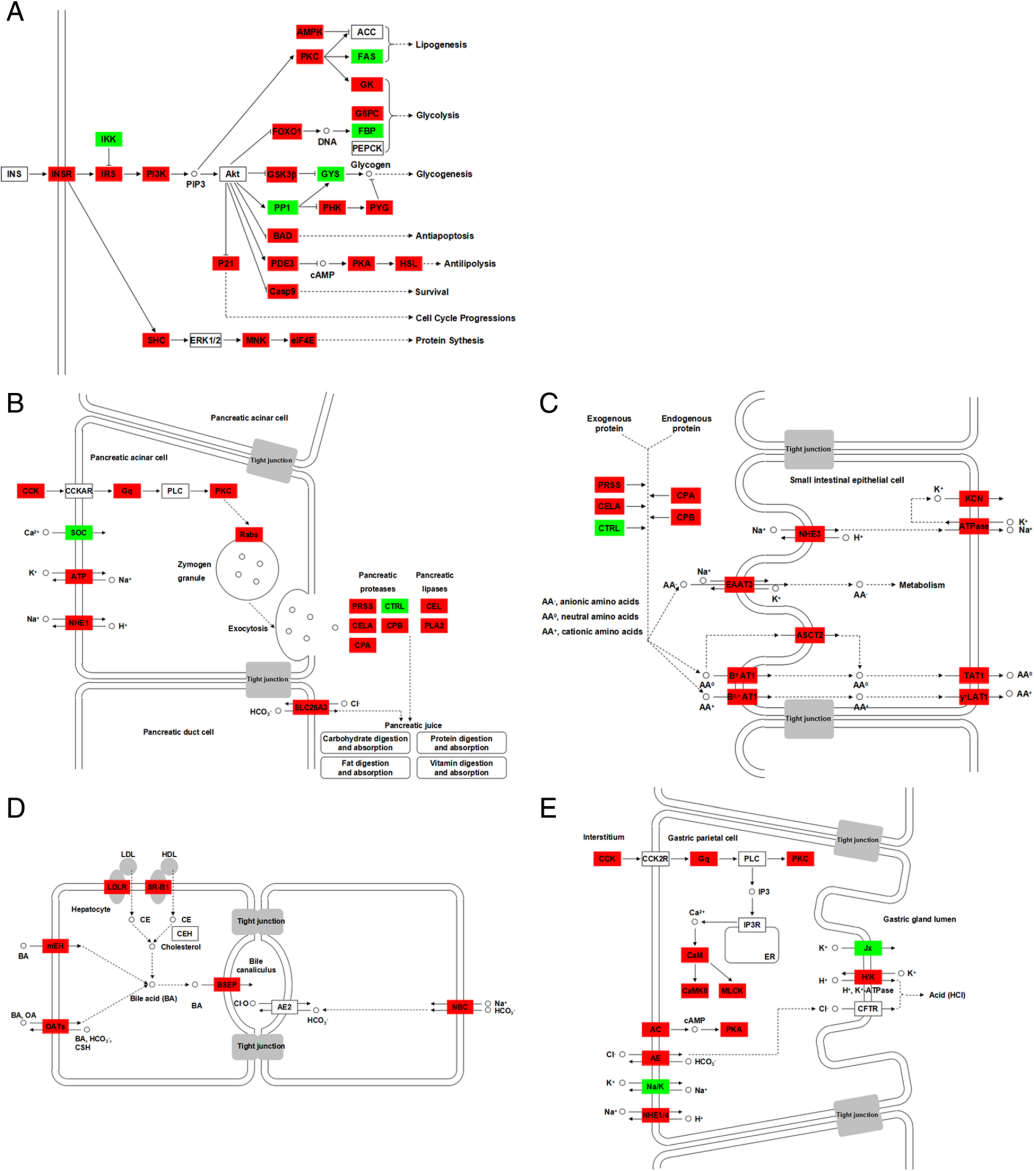
**Differentially expressed genes in digestion and metabolism pathway between grass carp before and after food habit transition from transcriptome analysis.** The most important pathways are insulin signaling and PPAR signaling **(A)**, pancreatic secretion **(B)**, protein digestion and absorption **(C)**, bile secretion **(D)**, gastric acid secretion **(E)**. The colors of rectangles were shaded according to the different expression (red: the mRNA expression levels of fish in Group C were significantly higher than those in Group A (FDR ≤ 0.001, the absolute value of log2[Ratio] ≥ 1); green: the mRNA expression levels of fish in Group C were significantly lower than those in Group A (FDR ≤ 0.001, the absolute value of log2[Ratio] ≥ 1)). All of these genes were not differentially expressed between Groups A and B.

In addition, expression of genes involved in digestion and metabolism were significantly different between fish before and after food habit transition, including increase of *hexokinase* (*GK*) and *glucose-6-phosphatase* (*G6PC*) involved in glycolysis (Figure 6A); *trypsin* (*PRSS*), *pancreatic elastase* (*CELA*), *carboxypeptidase A* (*CPA*), *carboxypeptidase B* (*CPB*), *bile salt-stimulated lipase* (*CEL*), *secretory phospholipase A2* (*PLA2*) involved in protein digestion (Figure 6B); *solute carrier family 1 member 1* (*EAAT3*), *solute carrier family 1 member 5* (*ASCT2*), *solute carrier family 6 member 19* (*B*^*0*^*AT1*), *solute carrier family 7 member 9* (*B*^*0,+*^*AT1*), *solute carrier family 16 member 10* (*TAT1*), *solute carrier family 7 member 7* (*y*^*+*^*LAT1*) involved in protein metabolism and absorption (Figure 6C); *microsomal epoxide hydrolase* (*mEH*), *solute carrier family 22 member 7* (*OATs*), *ATP-binding cassette subfamily B (MDR/TAP) member 11* (*BSEP*) involved in bile secretion (Figure 6D); *solute carrier family 26 member 7* (*AE*) and *H*^*+*^*/K*^*+*^*-exchanging ATPase alpha polypeptide* (*H/K-ATPase*) involved in gastric acid secretion (Figure 6E); *carnitine O-palmitoyltransferase 1* (*CPT-1*), *carnitine O-palmitoyltransferase 2* (*CPT-2*), *sterol carrier protein 2* (*SCP-x*), *acetyl-CoA acyltransferase 1* (*Thiolase B*) and *long-chain acyl-CoA synthetase* (*ACS*) involved in fatty acid oxidation and transport (Figure 4B).

**Figure 7:**
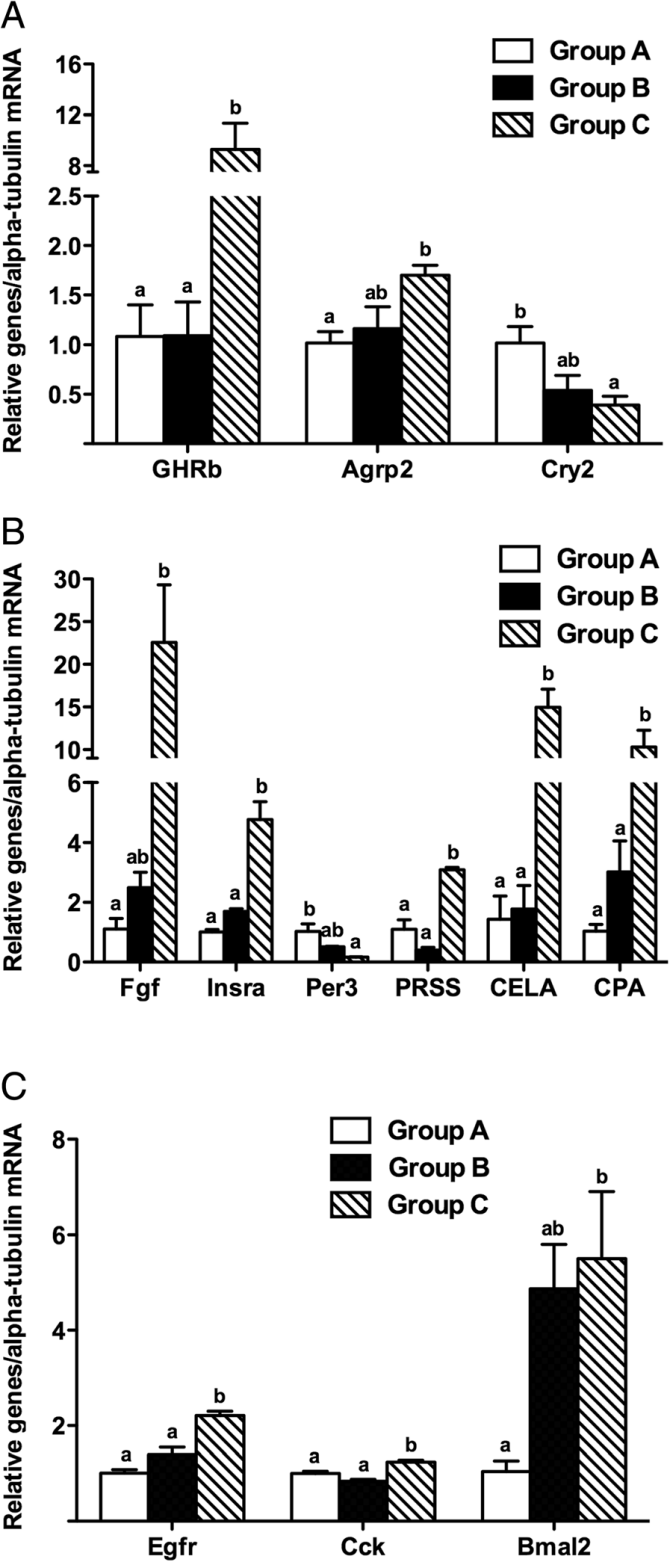
**Validation of differentially expressed genes with Real-time RT-PCR.** The relative mRNA abundance of differentially expressed genes in brain **(A)**, liver **(B)** and gut **(C)** were determined by Real-time RT-PCR. Data are presented as mean ± standard error (n = 4). A value followed by a superscript differs significantly from all other values not followed by the same superscript in the same kind of columns based on one-way analysis of variance (ANOVA) followed by the post hoc test (P < 0.05).

Furthermore, the importance of the differentially expressed genes indicated above was further supported by the identification of significant alternative splicing in these genes. 56% of these differentially expressed genes had alternative splicing events. We also found that gene structure of these differentially expressed genes in grass carp were different from those in zebrafish, suggesting that the unique gene structure of grass carp might contribute to its unique food habit transition (Figure 2). We used Real-time RT-PCR to confirm the important differential expression genes related to the food habit transition in grass carp. The data obtained were consistent with those obtained from the transcriptome sequencing and DGE analysis (Figure 7).

## Discussion

Although grass carp is an ecologically appealing model of vertebrate herbivore, little is known about the genes and biological mechanisms of herbivory formation and food habit transition in grass carp. In this study, by profiling the transcriptomes of grass carp before and after food habit transition, we identified differentially expressed genes potentially influencing the food habit transition from carnivory to herbivory, including those affecting cell proliferation and differentiation, appetite control, circadian rhythm, and digestion and metabolism. Real-time RT-PCR confirmed the differential expression in selected genes. Gene structures of several differentially expressed genes in grass carp were different from those in zebrafish. We also found numerous alternative splicing and novel transcript related to the food habit transition of grass carp. These differences together might account for the formation of herbivore, and the higher intake and utilization of plant feedstuff in grass carp.

### Gut growth and differentially expressed genes involved in cell proliferation and differentiation, and digestion and metabolism

Fish after food habit transition to herbivory (Group C) had significantly higher relative length of gut than fish before food habit transition (Group A) or those without food habit transition fed with chironomid larvae (Group B). It is suggested that longer gut could enable fish to achieve higher growth rates on plant materials, which have a relatively poor nutritional quality [18]. The digestive system plays an essential role in vertebrate physiology as the site of nutrient digestion and absorption [22]. Previous studies demonstrated that the time of exposure of ingested food to proteolytic enzymes rises with increasing gut length in herbivorous fish, therefore many herbivorous fish have long coiled digestive tract [23]. Furthermore, we observed higher expression of several growth factors, their receptors and downstream signaling molecular involved in cell proliferation and differentiation in Group C, including *GHRb*, *Egfr*, *Fgf*, *Fgfbp1*, *Insra*, *Irs2*, *Jak*, *STAT*, *PI3K*, *PKC* [24-31]. Previous study has reported that exogenous growth hormone stimulates structural and functional intestinal adaptation in rats [32]. GH receptors are present throughout the human gastrointestinal tract [33] and transgenic mice that overexpressed GH have higher total body weights and heavier small intestines than the control (nontransgenic) mice [34]. The intestinal EGF and EGFR are involved in the processes of gastrointestinal cell proliferation, differentiation, and migration [35]. Fibroblast growth factors have also been implicated in proliferation regulation in the gut [36]. Our results suggested that the up-regulation of these genes in grass carp after food transition might lead to increased cell proliferation and differentiation, contributing to the gut growth, food habit transition from carnivory to herbivory, and increase of intake and utilization of plant feedstuff in grass carp (Figure 3).

In addition, several genes involved in digestion and metabolism were significantly increased in grass carp after food habit transition, including *PRSS*, *CELA*, *CPA*, *CPB*, *CEL*, *PLA2* involved in protein digestion [37-39]; *EAAT3*, *ASCT2*, *B*^*0*^*AT1*, *B*^*0,+*^*AT1*, *TAT1*, *y*^*+*^*LAT1* involved in protein metabolism and absorption [40]; *mEH*, *OATs*, *BSEP* involved in bile secretion [41]; *AE* and *H/K-ATPase* involved in gastric acid secretion [42]; *GK* and *G6PC* involved in glycolysis; *CPT-1*, *CPT-2*, *SCP-x*, *Thiolase B* and *ACS* involved in fatty acid oxidation and transport [43] (Figure 6). It is suggested that longer gut could enable fish after the food transition to achieve higher growth rates on plant materials through increased digestion and metabolism, such as better protein digestion with increased *PRSS*, *CELA* and *CPA* expressions, better food digestion with enhanced bile and gastric acid secretion, and better protein absorption with improved amino acid transportation.

### Differentially expressed genes involved in appetite control

In the present study, grass carp had free access to food 24 h a day. Fish fed with low nutritional plant diets (Group C) had higher growth than those fed with high nutritional animal diets (Group B), therefore grass carp after food habit transition to herbivory could consume more food per day. Previous studies provide a framework for understanding the regulation of food intake in mammals and fish. Peripheral signals such as leptin from adipocytes, insulin from endocrine pancreas, cholecystokinin and peptide YY from gastrointestinal tract are incorporated in the hypothalamus to generate orexigenic (such as NPY and ghrelin) or anorexigenic (such as α-melanocyte stimulating hormone derived from proopiomelanocortin) signals [44]. We observed higher expression of orexigenic genes (*Agrp2*, *GABAra*), and lower expression of anorexigenic genes (*Npy y2*, *Drd1*) in brain of grass carp in Group C than those in Group A. Moreover, the expression of anorexigenic genes (*Leptin*, *Cck*, *Insra*, *Irs2*, *Trhr1*) were increased in liver or gut of grass carp in Group C compared to those in Group A (Figure 4). These genes are well-established regulators of energy homeostasis and play important roles in determination of food intake [45-49]. The changes in gene expression suggested that grass carp after food habit transition to herbivory had increased appetite signal in brain. This agrees well with the results obtained in grass carp fed with plant food that appears to feed throughout the diet cycle [18]. This foraging strategy may represent an adaptation to herbivory and enable the grass carp to achieve high growth rates on plant materials.

CCK is released from the duodenum in response to the presence of digested food [50], potentially explaining the increased expression in fish after food habit transition. In addition, leptin appears to be a growth factor for normal small intestine [51], and the increased expression of *Leptin* in liver might stimulate the gut growth of grass carp after food transition. Herbivorous fish consume more food per day and have much longer gut than carnivorous and omnivorous fish [52,53]. Our previous study on food preference of mandarin fish, a piscivore, showed that dead prey fish feeders have decreased appetite [54], suggesting that the appetite control pathway plays an important role in food habit formation of fish. In addition, several genes involved in glutamategic synapase, calcium signaling and GABAergic synapase pathway, such as *EAAT*, *GAD*, *NMDAR*, *PKC*, *PLA2*, *mGluR5*, *PKA* and *NOS* [55,56], were increased in grass carp after food habit transition, which might contribute to its higher appetite.

### Differentially expressed genes involved in circadian rhythm

Previous studies demonstrated that the molecular mechanisms of circadian rhythm generation in zebrafish appear to be generally consistent with the mammalian model [57]. We identified homologs of the mammalian clock genes in grass carp. We found differential expression in several clock genes, including *Per1*, *Per3*, *Cry*, *Clock*, *Bmal2*, *Pdp*, *Dec*, *Fbxl3*, *nocturnin* between fish before and after food transition (Figure 5). These genes are known to be critical regulators of circadian rhythm [58-63], with the heterodimerization of CLOCK and BMAL1 proteins activating transcription of *Period* and *Cryptochrome* genes. The PER and CRY proteins form complexes that enter the nucleus, bind to the CLOCK:BMAL1 complex and inhibit transcription. The disruption of these genes could cause the reset of behavioral rhythmicity. Grass carp fed with plant diet spent longer time on feeding, and feed almost continuously for most of the diet cycle [18]. Taken together, changes in expression levels of these clock genes in grass carp might reset circadian phase of feeding to accommodate the food habit transition from carnivory to herbivory, because fish fed with plant diet consumes less dry matter per bite than those fed with animal diet. This result agrees with our previous research in mandarin fish, with the acquisition of novel food preference (dead prey fish) partly due to resetting of circadian phase [54].

## Conclusions

In summary, our results showed that grass carp after food habit transition from carnivory to herbivory had higher relative length of gut than those before transition and without transition. The food habit transition in grass carp might be due to enhanced gut growth, increased appetite, resetting of circadian phase and enhanced digestion and metabolism. Interaction of expression and alternative splicing in genes related to cell proliferation and differentiation, appetite control, circadian rhythm outputs, and digestion and metabolism might drive the formation of herbivory in grass carp.

## Methods

### Fish and sample preparation

The embryos of grass carp were obtained from Wuhan Academy of Agricultural Science and Technology (Wuhan, Hubei Province, China). Larvae were raised in tanks at 25 ± 2°C and fed with chironomid larvae *Chironomus tentans*. At 46 days post-hatch (dph) (body weight 0.39 ± 0.05 g, body length 28.05 ± 0.99 mm), 30 fish were randomly selected for sample collection as fish before food habit transition (Group A). And then the rest of the fish were randomly divided into two groups (n = 1000 for each group) fed with either chironomid larvae *Chironomus tentans* as fish without transition (Group B) or duckweed *Lemna minor* as fish after food habit transition to herbivory (Group C). Fish had free access to food 24 h a day and fed for 70 days. At 116 dph (body weight and body length for Group B were 2.97 ± 0.3 g and 53.96 ± 1.80 mm, respectively; those for Group C were 7.34 ± 1.43 g and 72.78 ± 6.15 mm, respectively), 30 fish were randomly selected from each group for sample collection. Total RNA was isolated from brain, liver and gut tissues using SV TRIzol Reagent (Invitrogen, Carlsbad, CA, USA) according to manufacturer's protocol. Equal amount of total RNA of each group were pooled for each tissue and used to construct the libraries for transcriptome analysis. The following formula were used to calculate three ratios: the ratio of gut length to body length (gut length/body length), the ratio of gut weight to body weight (gut weight/body weight) and the ratio of hepatopancreas weight to body weight (hepatopancreas weight/body weight) [23,64]. The animal protocol was approved by the Ethical Committee of Huazhong Agricultural University.

### Transcriptome library preparation and Illumina sequencing

The samples for transcriptome analysis were prepared using Illumina's kit following manufacturer's instructions (San Diego, CA, USA). Poly(A) mRNA was purified from total RNA using oligo-dT-attached magnetic beads. Paired-end cDNA libraries were sequenced using Illumina HiSeq2000 system. Image deconvolution and base calling were performed with the Illumina CASAVA v1.7. The reliability of the reads was 89.1% with average length of the reads at 90 bp. Clean reads were obtained by removing adaptor reads and low quality reads (Q ≤ 5), on which all following analysis are based. The library construction and sequencing were performed by Beijing Genomics Institute at Shenzhen (Shenzhen, China). To estimate expression levels and discover novel genes and transcripts, the RNA-Seq reads generated were mapped to the grass carp genome using the Short Oligonucleotide Analysis Package SOAPaligner/soap2 [65], up to five base mismatches were allowed in the genome alignment while up to two base mismatches were allowed in gene alignment. The reference genome and annotation data of grass carp were obtained from the State Key Laboratory of Freshwater Ecology and Biotechnology, Institute of Hydrobiology, Chinese Academy of Sciences, China.

### Alternative splicing and novel transcript predication

To identify all potential splice sites, we searched for three types of splice of site (Class I: GT-AG/CT-AC; Class II: GC-AG/CT-GC; and Class III: AT-AC/GT-AT) in the intronic regions. Alternative splicing events were classified into four basic types: exon skipping, intron retention, alternative 5’ splice site, alternative 3’ splice site. SOAPsplice [66] (with all default parameters) was used to detect the splice junction sites which give information about boundaries and combinations of different exons in a transcript. Then all splice junction sites of the same gene are used to distinguish type of its alternative splicing event. To detect novel genes in the putative intergenic region, we compared the reference gene models and the transcriptome, the potential gene models found in intergenic regions (200 bp away from upstream or downstream of genes) with lengths > 150 bp and average coverage > 2 were considered to be candidate of novel transcript.

#### Identification of differentially expressed genes

Gene expression levels were measured through short reads mapping in Reads Per Kb per Million reads (RPKM) [67]. Kyoto Encyclopedia of Genes and Genomes (KEGG) pathway analysis were then carried out in differentially expressed genes. To annotate the differentially expressed genes, we performed the BLASTx alignment (e-value < 0.00001) against protein databases such as NCBI, Swiss-Prot, KEGG and COG. SYBR Green Real-time RT-PCR was performed to validate the transcriptome data (Additional file 5). Alpha-tubulin was amplified in parallel as an internal control. There were four biological and three technical replicates respectively.

### Statistical analysis

We used FDR ≤ 0.001 and the absolute value of log2[Ratio] ≥ 1 as the threshold to judge the significance of gene expression difference. Statistical analysis was performed with SPSS13.0 software. Data normality and homogeneity of variances were analyzed. Results were presented as the means ± S.E. for each group. One-way analysis of variance (ANOVA) followed by the post hoc test were carried out to determine whether the differences between groups were significant (P < 0.05).

### Availability of supporting data

All the supporting data are included as additional files.

## Additional files

Additional file 1:
**Distribution statistics of reads mapped to reference genes.** AB, AL and AG indicate brain, liver and gut in Group A, respectively; BB, BL and BG indicate brain, liver and gut in Group B, respectively; CB, CL and CG indicate brain, liver and gut in Group C, respectively. Additional file 2:
**Distribution statistics of genes’ coverage.** AB, AL and AG indicate brain, liver and gut in Group A, respectively; BB, BL and BG indicate brain, liver and gut in Group B, respectively; CB, CL and CG indicate brain, liver and gut in Group C, respectively. Gene coverage is the percentage of a gene covered by reads. The value equals to ratio of the number of bases in a gene covered by unique mapping reads to number of total bases in that gene. Additional file 3:
**Alternative splicing prediction.** **(A)** Numbers of alternative splicing events and involved genes in the three groups. **(B)** Numbers of four major alternative splicing events and involved genes in the three groups. The x-axis represents types of alternative splicing events (AS Event). A: fish fed with chironomid larvae before food habit transition; B: fish fed with chironomid larvae without transition; C: fish fed with duckweed after food habit transition to herbivory. Additional file 4:
**Differentially expressed genes (DEG) analyzed by transcriptome sequencing.** AB, AL and AG indicate brain, liver and gut in Group A, respectively; BB, BL and BG indicate brain, liver and gut in Group B, respectively; CB, CL and CG indicate brain, liver and gut in Group C, respectively. The superscripts of each column represent the number of differentially expressed genes between groups. Additional file 5:
**Primer sequences for Real-time RT-PCR.**
