# Supplementary figures and images for: Transcriptome analysis of food habit transition from carnivory to herbivory in a typical vertebrate herbivore, grass carp Ctenopharyngodon idella
Source: BMC Genomics. 2015 Jan 22;16(1):15. doi: 10.1186/s12864-015-1217-x (PMC4307112; doi:10.1186/s12864-015-1217-x)

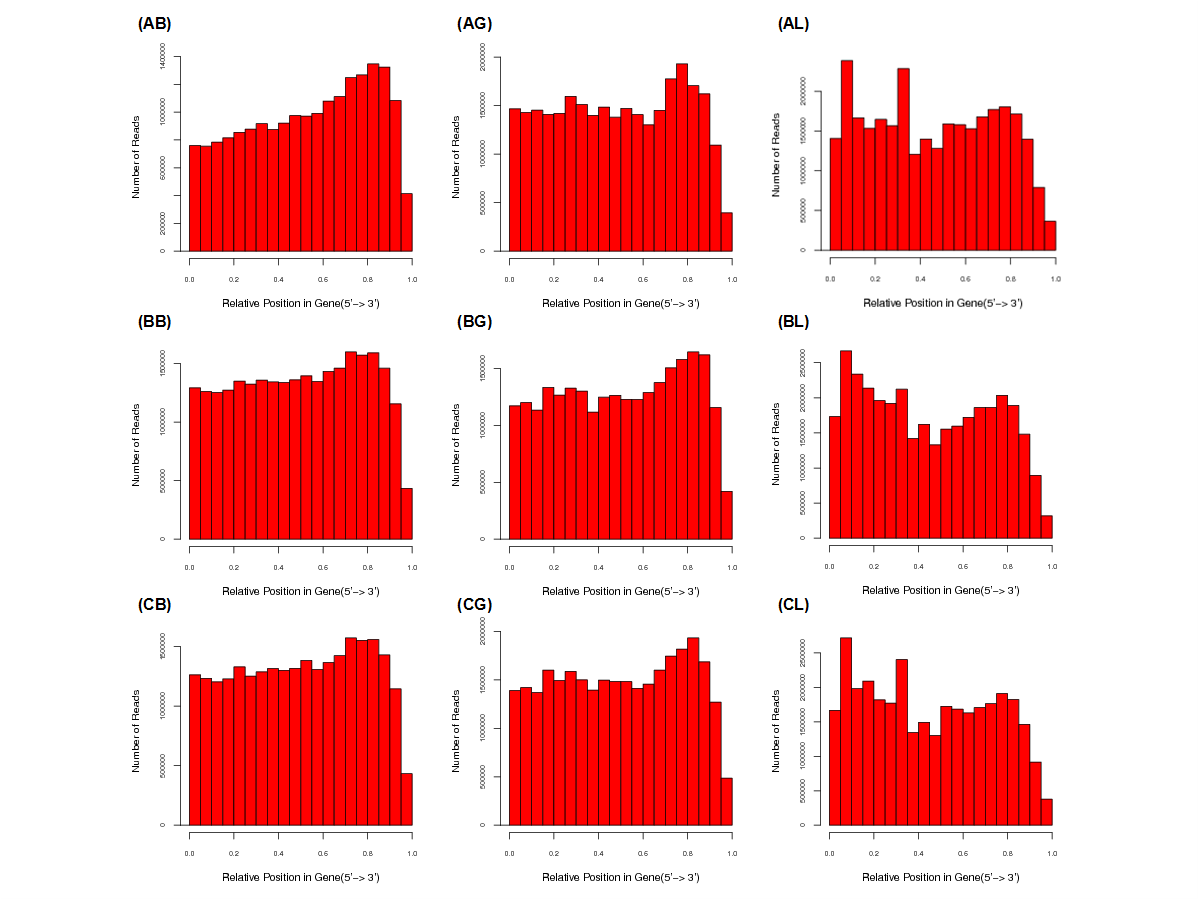

Supplement: Additional file 1: — Distribution statistics of reads mapped to reference genes. AB, AL and AG indicate brain, liver and gut in Group A, respectively; BB, BL and BG indicate brain, liver and gut in Group B, respectively; CB, CL and CG indicate brain, liver and gut in Group C, respectively. [file 12864_2015_1217_MOESM1_ESM.tiff]

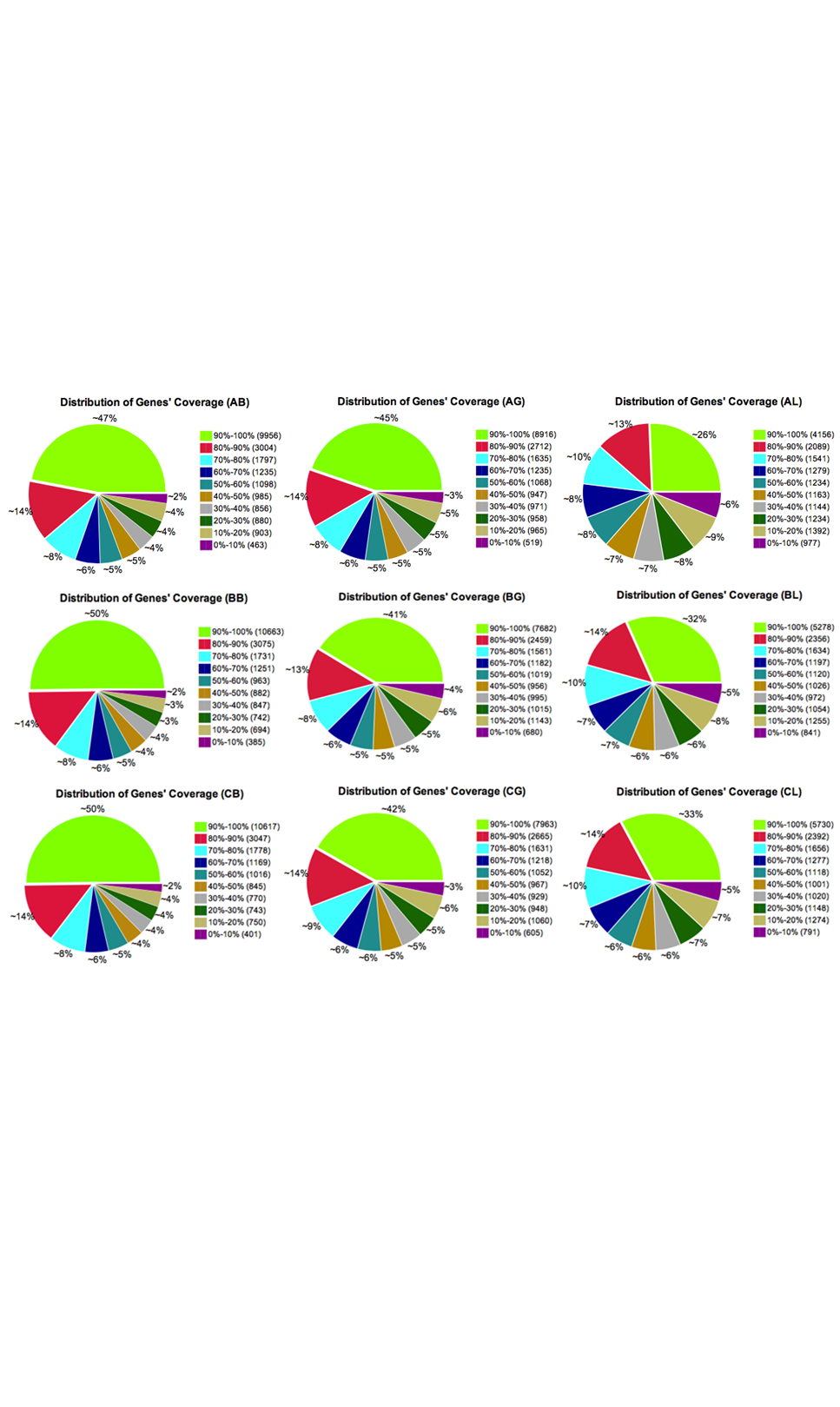

Supplement: Additional file 2: — Distribution statistics of genes’ coverage. AB, AL and AG indicate brain, liver and gut in Group A, respectively; BB, BL and BG indicate brain, liver and gut in Group B, respectively; CB, CL and CG indicate brain, liver and gut in Group C, respectively. Gene coverage is the percentage of a gene covered by reads. The value equals to ratio of the number of bases in a gene covered by unique mapping reads to number of total bases in that gene. [file 12864_2015_1217_MOESM2_ESM.tiff]

A

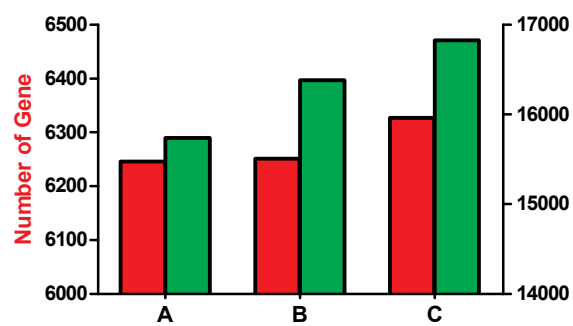

B

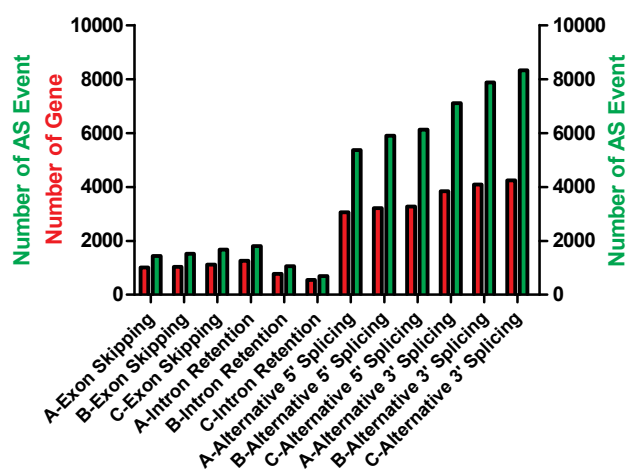

Supplement: Additional file 3: — Alternative splicing prediction. (A) Numbers of alternative splicing events and involved genes in the three groups. (B) Numbers of four major alternative splicing events and involved genes in the three groups. The x-axis represents types of alternative splicing events (AS Event). A: fish fed with chironomid larvae before food habit transition; B: fish fed with chironomid larvae without transition; C: fish fed with duckweed after food habit transition to herbivory. [file 12864_2015_1217_MOESM3_ESM.pdf]

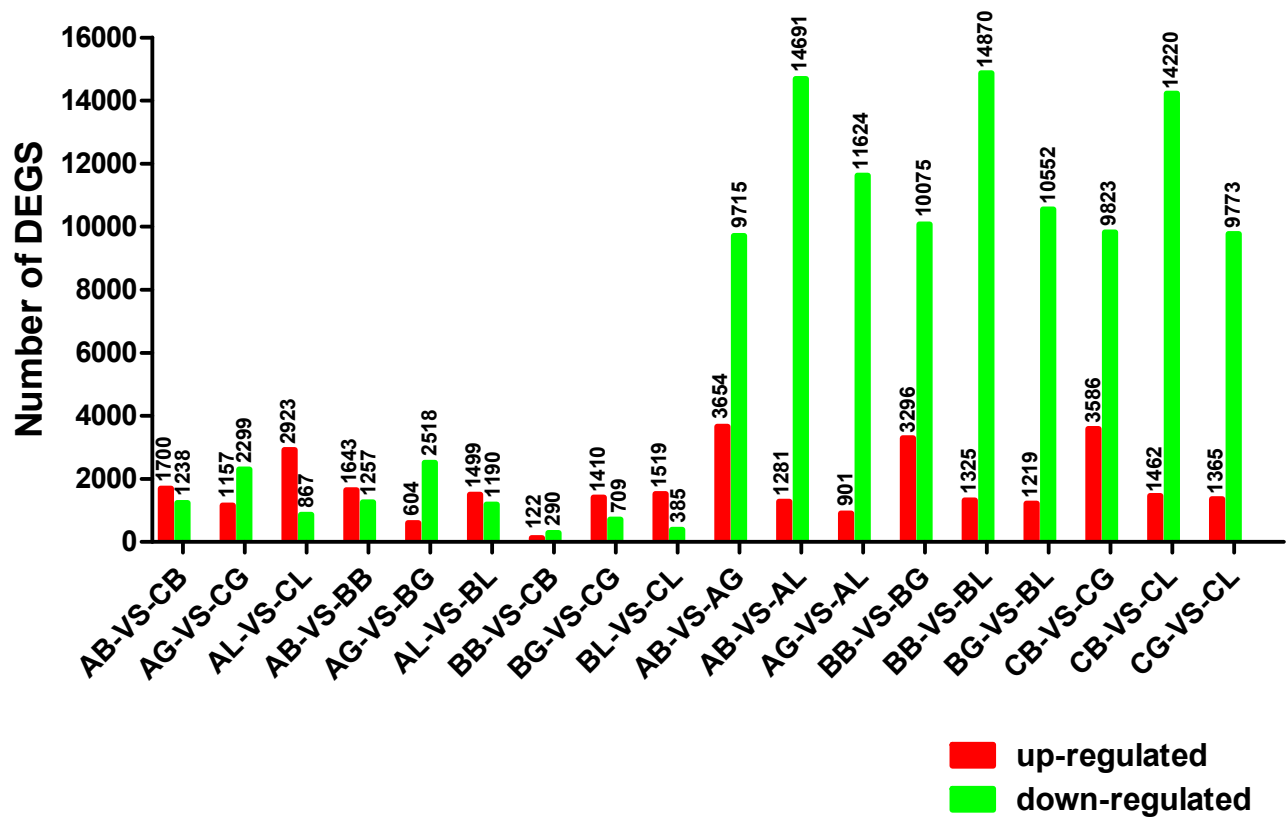

Supplement: Additional file 4: — Differentially expressed genes (DEG) analyzed by transcriptome sequencing. AB, AL and AG indicate brain, liver and gut in Group A, respectively; BB, BL and BG indicate brain, liver and gut in Group B, respectively; CB, CL and CG indicate brain, liver and gut in Group C, respectively. The superscripts of each column represent the number of differentially expressed genes between groups. [file 12864_2015_1217_MOESM4_ESM.pdf]
