# Supplementary material for: Transcriptome analysis of food habit transition from carnivory to herbivory in a typical vertebrate herbivore, grass carp Ctenopharyngodon idella
Source: BMC Genomics. 2015 Jan 22;16(1):15. doi: 10.1186/s12864-015-1217-x (PMC4307112; doi:10.1186/s12864-015-1217-x)
Supplement: Additional file 5: — Primer sequences for Real-time RT-PCR. [file 12864_2015_1217_MOESM5_ESM.docx]

**Additional file 5. Primer sequences for Real-time RT-PCR.**

| **Tissue** | **Gene name** | **Sequence of the primers (5′-3′)** |
| --- | --- | --- |
| Internal control gene | Alpha-tubulin | Forward: CTGAGCAACACTACAGCCATT |
|  |  | Reverse: CTTCATCCTCTTCTCCGACAC |
| Brain | GHRb | Forward: AATAAGCCAAACCTACCTACG |
|  |  | Reverse: TTTCCAGTCCACCCCATCTA |
|  | Agrp2 | Forward: CACCACAGCTCTGCCATAAC |
|  |  | Reverse: CTGGGCTTAGGTCTCACCAC |
|  | Cry2 | Forward: AGCAGGATTCAGACCATCACCC |
|  |  | Reverse: CTTACGCAGACGACGCCTTTGT |
| Liver | Fgf | Forward: TGACTGAGTTCTCCCGTTCC |
|  |  | Reverse: GCGTTTGTGCTGTTTAGTTGAT |
|  | Insra | Forward: ACTTTCTCCTGTTCCGTGTC |
|  |  | Reverse: GTAGTTGTCCTCCACCGAGT |
|  | Per3  PRSS | Forward: AACACACGCTCAAAAATACA |
|  |  | Reverse: CCACTCAATCCCTGAACCTT |
|  |  | Forward: ATGGTATTGTGTCCTGGGGTT  Reverse: TAGTTGCTGCTTATGGTGTCG |
|  | CELA  CPA | Forward: GCATTGGCAGCCGAACTTACA |
|  |  | Reverse: CATTGGTCCAGAGGCGTCCCC  Forward: GCAACTTCAAATCCTTCATCTCC  Reverse: CGTAGTAACCAGTGTCACGCAAC |
| Gut | Egfr | Forward: TGGAGCTTTTGGCACTGTGT |
|  |  | Reverse: CTGGGTGATAAGTTGGACGG |
|  | Cck | Forward: CATAGTGGAACACACACGCC |
|  |  | Reverse: GGCCAAAATCCATCCATCCC |
|  | Bmal2 | Forward: CCAGGAAACGGAAAGGCAGCAT |
|  |  | Reverse: GGTTACAGGTGGGAATCATCGC |
